# Zebrafish Reproduction: Revisiting In Vitro Fertilization to Increase Sperm Cryopreservation Success

**DOI:** 10.1371/journal.pone.0021059

**Published:** 2011-06-16

**Authors:** Mary Hagedorn, Virginia L. Carter

**Affiliations:** 1 Smithsonian Conservation Biology Institute, Smithsonian National Zoological Park, Washington, D.C. United States of America; 2 Hawaii Institute of Marine Biology, University of Hawaii, Kaneohe, Hawaii, United States of America; Temasek Life Sciences Laboratory, Singapore

## Abstract

Although conventional cryopreservation is a proven method for long-term, safe storage of genetic material, protocols used by the zebrafish community are not standardized and yield inconsistent results, thereby putting the security of many genotypes in individual laboratories and stock centers at risk. An important challenge for a successful zebrafish sperm cryopreservation program is the large variability in the post-thaw in vitro fertilization success (0 to 80%). But how much of this variability was due to the reproductive traits of the in vitro fertilization process, and not due to the cryopreservation process? These experiments only assessed the in vitro process with fresh sperm, but yielded the basic metrics needed for successful in vitro fertilization using cryopreserved sperm, as well. We analyzed the reproductive traits for zebrafish males with a strict body condition range. It did not correlate with sperm volume, or motility (P>0.05), but it did correlate with sperm concentration. Younger males produced more concentrated sperm (P<0.05). To minimize the wastage of sperm during the in vitro fertilization process, 10^6^ cells/ml was the minimum sperm concentration needed to achieve an in vitro fertilization success of ≥ 70%. During the in vitro process, pooling sperm did not reduce fertilization success (P>0.05), but pooling eggs reduced it by approximately 30 to 50% (P<0.05). This reduction in fertilization success was due not to the pooling of the females' eggs, but to the type of tools used to handle the eggs. Recommendations to enhance the in vitro process for zebrafish include: 1) using males of a body condition closer to 1.5 for maximal sperm concentration; 2) minimizing sperm wastage by using a working sperm concentration of 10^6^ motile cells/ml for in vitro fertilization; and 3) never using metal or sharp-edged tools to handle eggs prior to fertilization.

## Introduction

In the past decade, laboratories around the world have produced tens of thousands of mutant, transgenic, and wild-type zebrafish lines. Maintaining all of these valuable genotypes is expensive, risky, and beyond the capacity of even the largest stock centers. Our long-term goal is to preserve genetic resources from aquatic model organisms, specifically zebrafish that are vital to advancing biomedical research and knowledge. Although conventional cryopreservation is a proven method for long-term, safe storage of genetic material, protocols used by the zebrafish community are not standardized and yield inconsistent results, thereby putting the security of many genotypes in individual laboratories and stock centers at great risk. A systematic approach based on fundamental cryobiological principles is essential to improving post-thaw fertilization and assuring the security of wild-type, mutant, and transgenic zebrafish sperm. However, little basic cryobiological data exist to permit methodical, orderly preservation, and use of this germplasm**.**

The cryopreservation needs of zebrafish stock centers and individual laboratories differ. Stock centers require rapid high-throughput (defined as using rapid, quantifiable and often bulk methods) and biosecure technologies to maximize number of samples preserved over time. In contrast, individual laboratories need preservation options that are specific to their particular local equipment, personnel, and space constraints. There is an immediate need to improve the preservation of aquatic research model organisms, especially zebrafish. In the past decade, laboratories have created >20,000 mutant, transgenic, and wild-type fish lines that are being used extensively to address high priority issues in toxicology, embryology, genetics, drug development, and human diseases [1]. The physical and scholarly capacity of this resource is enormous, but is at significant risk because the ability to maintain these living, whole animal collections is logistically complex, costly, and requires vast amounts of space.

Although cryopreservation is a proven method for long-term maintenance of genetic material, current protocols for fish are not standardized and yield inconsistent results, threatening the efficacy of large-scale genetic screening and stock centers. Previous reports have dealt with sperm cryopreservation in >200 fish species [2], [3] with the most common observation from these publications being the inconsistency in the post-thaw results. The most serious challenge is that the zebrafish community continues to rely on a single technique and protocol developed more than 25 years ago [4] that has been adapted for all zebrafish [5]. Several laboratories have modified this original protocol, but not on basic principles of cryobiology – where there is a need to clearly understand the biophysical properties of a cell to allow optimal freezing and post-thaw survival. In a few cases, modifications of the original ‘Harvey Method’ [6], [7] have allowed adequate survival of stored zebrafish sperm in individual laboratories [8]. However, there is no accompanying knowledge to explain why these slightly modified protocols work in one laboratory, but not another. Most importantly, there is insufficient scientific rigor to allow generating better protocols to permit a diversity of zebrafish genotypes to be banked simultaneously on a large-scale in stock centers – simply to catch-up to safeguarding this enormous resource.

In order to move forward and standardize our practices, we have examined some basic reproductive traits of zebrafish sperm important for successful cryopreservation. One of the biggest challenges that still remains today for a successful zebrafish sperm cryopreservation program is the variability in the post-thaw *in vitro* fertilization success (0 to 80%); [4]). Part of this variability may be due condition factors of the fish [9] prior to harvesting the sperm, because it is clear that good nutrition matters for a successful cryopreservation process [10]. Yang et al [10] specifically noted that zebrafish in poor nutritional health have poor post-thaw reproductive traits compared to their high nutritional health counterparts. The Zebrafish International Resource Center (ZIRC) has a standardized zebrafish diet and holding facilities that maintains the animals under low stress and in excellent reproductive health (www.zfin.org). These standard practices and strict condition factors of weight and standard length were the starting point for these studies.

Because variability in the post-thaw fertilization success is the primary concern for stock centers, this paper addressed whether some of this variability might be present in the fresh sperm and reproductive traits, prior to the cryopreservation process. We examine traits such as the correlation of condition factor with mean volume, concentration and motility of squeezed ejaculate, the minimum sperm concentration needed to achieve an *in vitro* fertilization success of >60%, and various male and female factors that may play a role in the *in vitro* fertilization process. All of these factors become important when considering high-throughput cryopreservation scenarios for zebrafish because how often you can harvest germplasm, its concentration and the minimum concentration needed for *in vitro* fertilizations all become time-limited commodities for resource centers.

## Methods

### Maintenance of Animals

Fish were housed in standard microcosms (Aquatic Ecosystems, Apopka, FL) that have independent, water, temperature and waste management with sensors on each rack to constantly monitor the pH, temperature and conductivity of the water. ZIRC has prepared detailed user manuals that describe standard operating procedures [5]. We followed their recommended facilities operations including care and maintenance of adults, breeding and obtaining gametes and embryos, record keeping, receiving fish from other laboratories, quarantine and other procedures relating to disease control, and euthanasia. Briefly, AB wild-type fish were obtained from stocks at ZIRC at approximately 4 months of age. They were maintained in recirculating dechlorinated systems at 26 to 28°C with an artificial light cycle (14 h light: 10 h dark). Feeding schedule consisted of twice-daily provision of live brine shrimp (*Artemia* nauplii) and “Master Mix” dry food (a combination of Nelson's Silver Cup Tropical No. 1, Spirulina Flake, Golden Pearl and Cyclopeeze), see Westerfield [5] for details. Fecal material and other debris was flushed form the tanks according to the design of the microcosms, while algae was cleaned from sides and filters changed weekly. All care and welfare for the animals met NIH animal care standards. Full details of the study approval are listed with the Smithsonian CRC-IACUC (approval ID #06-19) and the University of Hawaii, Hawaii Institute of Marine Biology IACUC (protocol ID# 06-022).

### Collection of Germplasm

Gentle squeezing of males and females was used to obtain mature eggs and sperm. The afternoon prior to squeezing, males and females were removed from group tanks and placed into divided breeder tanks with one male on a side and 5–7 females on the other. These breeder tanks contained artificial plants to encourage spawning readiness in the females. On occasions when only males were needed for squeezing, males were simply separated from females the night before and housed in a separate tank as a group.

For the squeezing procedure, we used the methods described in Westerfield [5]. Briefly, gravid animals were immersed in a solution of tricaine methane sulfonate (MS-222) made according to Westerfield [5] until gill movements have slowed (∼30 sec). To collect sperm, males were rinsed with clean aquarium water, excess water was removed by placing fish on a Kim wipe, so that standard length and weight could be taken and recorded. Fish were then placed in a damp sponge with the dorsal surface down. While viewed under a dissecting microscope, the anal fin area was dried and gentle pressure was exerted using forceps to squeeze both sides of the fish from just posterior to the pectoral fin to a point just anterior to the anal fin. The sperm was collected with a 10 µl calibrated capillary tube, amount recorded and then placed into an Eppendorf tube on ice to await more sperm, if pooling experiments were done. Unless specified otherwise, all solutions were made in a chilled Hanks balanced salt solution (HBSS) at 300 to 305 mOsm/kg (0.137 M NaCl, 5.4 mM KCl. 1.3 mM CaCl. 1.0 mM MgSO_4_, 0.25 mM Na_2_HPO_4_, 4.2 mM NaHCO_3_ and 5.55 mM glucose, pH 7.2) to maintain the sperm and prevent activation following the methods of Yang and Tiersch, [11] and Jing et al., [12]. After squeezing, males were returned to a recovery tank for observation. Sperm was then diluted into HBSS to appropriate concentrations depending on the needs of each experiment and was held on ice until use in a fertilization trial. Males were squeezed first so that sperm was ready for fertilizations when eggs were obtained.

To collect eggs, females were anesthetized (as above), rinsed in clean aquarium water, gently dried and length and weight taken and recorded. Each female was placed on its side in a 35 mm plastic Petri dish. Gentle pressure was applied with one finger on the ventral side of the fish just below the pectoral fins and one finger on the dorsal side of the fish, with slight movement of the fingers back towards the pelvic fins. Eggs were expressed through the cloacal opening and held dry or in HBSS buffer (depending on the needs of the experiment) in 35-mm Petri dish and covered prior to mixture with sperm. After the eggs were collected, the females were placed into a recovery tank for observation.

### Experiment 1 - Body Condition and Sperm Quality

While it is accepted that standard husbandry practices should be followed for maintaining good stocks of zebrafish, we wanted to directly examine the effect of fish condition on sperm quality. Overall relative wellness of fish is expressed by the condition factor, abbreviated “K”, which compares the weight of the fish to its standard length by the following equation: K^ = ^100,000 x weight/(standard length)^3^ [10]. Individually identified male zebrafish were squeezed as described above. Standard length and weight were measured for each fish (N = 35), and their condition factor was calculated. Sperm volume, motility and concentration were recorded and assessed in comparison to condition factor for each fish. Motility was determined visually on a phase microscope (Olympus BX41) by measuring the mean percent progressive motility. To measure the motility, two µl of sperm at 10^7^ cells/ml were placed onto the surface of a slide, 18 µl of deionized water was added to activate the sperm, the drop was gently mixed on the slide, and the motility measured within 5 to 10 sec of mixing. The slide was moved to assess at least 3 full frames of sperm motility and estimated at <10, 25, 50, 75 or >90% motility.

### Experiment 2 - Smallest Reproductive Unit

Because zebrafish males typically produce a small ejaculate volume (∼1 µl) [4], the packaging and the number of straws or cryovials, and the time needed for personnel to process these samples to re-establish a line becomes an important consideration for high-throughput cryopreservation. Therefore, the minimum concentration of sperm needed to yield successful fertilization, defined for these studies as >60% measured at 24 hours, was determined. For *in vitro* fertilization, a metal spatula was used to divide a clutch into groups of ∼20 to 30 eggs (except for Experiment 5). Males were squeezed, as described above, and sperm from several males was pooled to produce a final volume of at least 8 µl/trial. This sample was diluted to ∼1×10^9^ cells/ml as measured on a hemocytometer, then portions diluted with HBSS to yield 10^8^, 10^7^, 10^6^,10^5^ and 10^4^ cells/ml. These diluted samples were used in standard *in vitro* fertilization protocols. Sperm (40 µl) at the various concentrations was added to a group of eggs held dry in a dish, 360 µl of 0.22 µm-filtered aquarium water (∼37 mOsm) was added to the eggs and sperm and gently mixed. This yielded a final working concentration of 10^8^ (N = 5), 10^7^(N = 21), 10^6^(N = 26), 10^5^(N = 25), 10^4^ (N = 25), and 10^3^ (N = 16) sperm to initiate fertilization in the dishes. We use the term “working fertilization concentration” to mean the concentration of sperm surrounding an egg to initiate the fertilization process in the dish. After 5 min, the mixture was topped up with 5 ml of methylene blue-treated embryo medium [5], cultured at 28.5°C and the fertilization success was checked after 1, 4 to 6 and 24 h. For all fertilizations in subsequent experiments, a working concentration of 10^6^ cells/ml was used.

### Experiment 3 - Individual Versus Pooled Gametes During Fertilization

One of the greatest problems for high-throughput cryopreservation is the variability in post-thaw fertilization success; however, an important factor may be differences in individual males and females that affect the outcome. Pooling of gametes might be considered an option to reduce variability in male and female gamete fitness. Therefore, we examined whether pooling might interfere with fertilization success, and addressed this by testing various *in vitro* fertilization combinations. Specifically, a clutch of eggs was divided into four parts for 4 treatments containing: 1) an individual female's oocytes and an individual male's sperm (N = 36 trials); 2) an individual female's oocytes and pooled sperm from at least 5 males (N = 58 trials); 3) pooled oocytes from 3 females and an individual male's sperm (N = 62 trials); and 4) pooled oocytes from 3 females and pooled sperm from at least 5 males (N = 49). Fertilization success was assessed at 1, 4 and 24 h.

### Experiment 4 - Effect of Holding Gametes on Fertilization Success

Gamete holding time was examined to determine whether it affected i*n vitro* fertilization success. First, pooled samples of sperm from 5 males were held on ice for either 1 h (N = 10 fertilization trials) or 2 h (N = 10 fertilization trials) prior to combining with freshly squeezed eggs from individual females. The pooling of female eggs was complicated by two additional variables, the effects of drying and the holding time. To examine dehydration versus pooling, eggs from individual females (N = 16) or pooled females (N = 4) were held dry or wet in 40 µl of HBSS in a 35 mm Petri dish for 2 min, and then the HBSS was removed prior to fertilization with the pooled sperm (N = 10 males). To examine holding time versus pooling, the eggs from individual females (N = 9) were divided into three equal portions. One portion was fertilized immediately with pooled sperm (N = 10 males), while the other portions were held dry or wet in 40 µl of HBSS in a 35 mm held for 5 min, and then the HBSS was removed prior to fertilization with the pooled sperm. Fertilization success was assessed at 1, 4 and 24 h.

### Experiment 5 - Egg Handling

The fertilization success from eggs moved with a metal spatula (standardly used for all the *in vitro* fertilizations in this study and in most laboratories and resource centers) was compared against Teflon-coated spatulas. Eggs from individual females (N = 10) were initially divided in half with the Teflon coated spatula, then each half was divided again with either the Teflon coated spatula or the metal spatula. Each quarter was moved into separate dish with the assigned spatula and covered in 40 µl of HBSS. One group of Teflon-moved and one group of metal-moved eggs were allowed to sit for 10 minutes before fertilization, while the remaining two groups were immediately fertilized by removing the HBSS and fertilizing as described above with pooled sperm (N = 5 males). Fertilization success was assessed at 1, 4 and 24 h.

### Data analysis

All data analysis in this study was performed using Graphpad Prism 5.0 (San Diego, CA) and Microsoft excel (version 2007). Correlation analysis, t-test and ANOVA (with a Neuman-Keuls post-test or Tukey's Multiple Comparison Test) were used on various data sets; these tests were identified specifically in the results when reporting the P-value.

## Results

### Experiment 1 - Body Condition Correlates with Sperm Concentration

**Figure 1:**
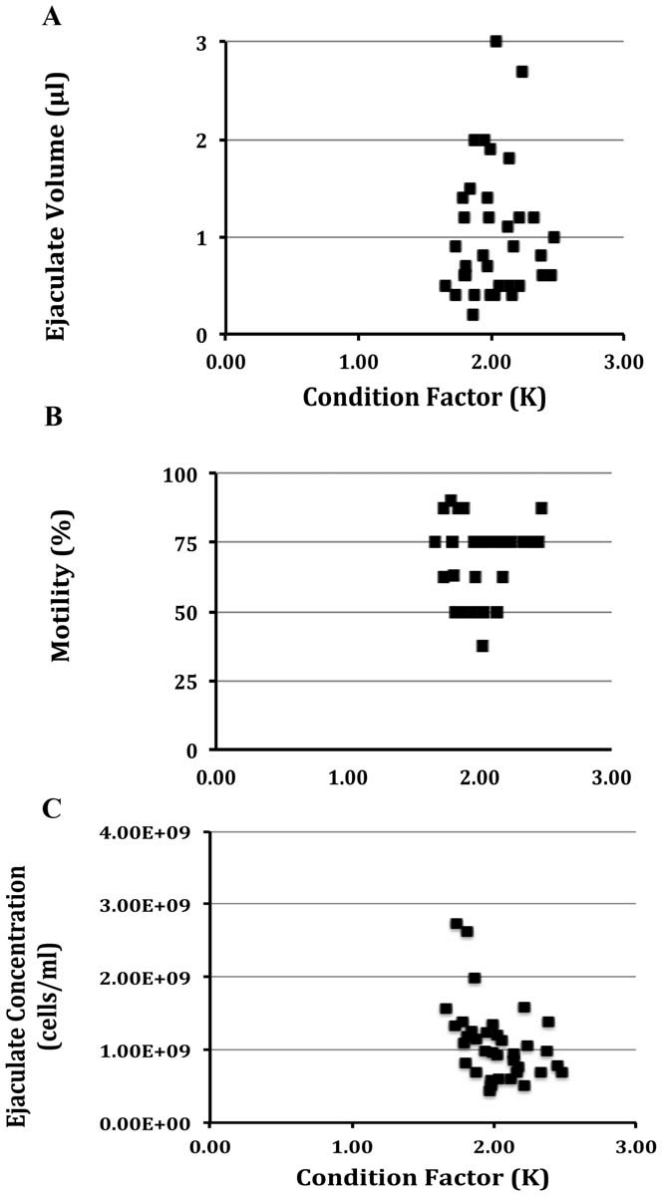
Reproductive traits and body condition. Male zebrafish (N = 35) were squeezed and their body condition (K^ = ^100,000 x weight/(standard length)^3^ ) determined and correlated with characteristics of the undiluted ejaculated. **A)** Body condition (K) did not correlate with sperm volume or **B)** motility, but **C**) increasing body condition (K) correlated with a decreasing sperm concentration. Analysis suggested that there was no correlation between body condition and sperm motility, nor body condition and ejaculate volume (Fig. 1 A and B; P>0.05, Linear regression). However, paradoxically, fish with increasing body condition, produced less concentrated sperm (Fig. 1C; P<0.05). In fact, the most extreme body condition values (1.73 and 2.47) demonstrated a 75% decrease in sperm concentration.

The condition of the animal can affect cryopreservation, but it was not known how it contributed to the variability in reproductive traits. The condition factor versus the sperm volume, motility and concentration were plotted (Fig. 1). Correlation analysis suggested that there was no correlation between body condition and sperm motility, nor body condition and ejaculate volume (Fig. 1 A and B; P>0.05). However, paradoxically, fish with increasing body condition, produced less concentrated sperm (Fig. 1C; P<0.05). In fact, the most extreme body condition values (1.73 and 2.47) demonstrated a 75% decrease in sperm concentration, meaning the smaller and younger fish produced much more concentrated sperm, and this factor may be an important consideration when considering which animals to choose for sperm donation.

### Experiment 2 - In Vitro Success is Determined by Sperm Concentration

**Figure 2:**
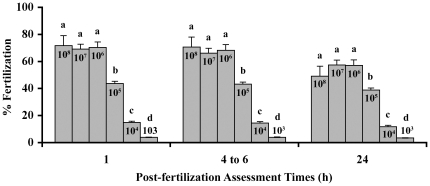
Minimal sperm needed for maximal in vitro fertilization. To test the optimal sperm concentration for zebrafish *in vitro* fertilization, various working sperm concentrations were assessed. These test concentrations (cells/ml) included 10^8^ (N = 5), 10^7^(N = 21), 10^6^(N = 26), 10^5^(N = 25), 10^4^ (N = 25), and 10^3^ (N = 16) sperm to initiate fertilization in the dishes. The means were significantly different (P<0.05) between the groups that have different letters (ANOVA). This determined that 10^6^ cells/ml was the minimal *in vitro* sperm concentration needed to achieve maximum fertilization success, below this concentration the fertilization success declined.

The minimum concentration of sperm needed for *in vitro* fertilization in zebrafish was unknown. In this experiment, a working sperm concentration of 10^6^ cells/ml of fresh sperm produced a mean fertilization success of 70% after 24 h of development (Fig. 2). Increasing the sperm concentration used for the *in vitro* methods did not increase the mean fertilization success (P<0.05; One way analysis of variance with Tukey's Multiple Comparison Test), but using less sperm (10^5^ to 10^3^ cells/ml) significantly decreased the mean fertilization success to 39, 12 and 3%, respectively (P<0.05; One way analysis of variance with Tukey's Multiple Comparison Test). The mean reproductive traits of the males used in experiments 1 and 2 were summarized in Table 1.

**Table 1:** Mean (± SEM) Reproductive Characteristics of Male Zebrafish.

Weight = 364.8±16.8 mg
Standard Length = 2.8±0.5 cm
Condition Factor = 2.02±0.04*
Squeezed Ejaculate:
Mean volume = 1.01 µl
Concentration = 1.1×10^10^±8.8×10^8^ cells/ml
Motility = 69%±2.30 @ 40 mOsm
Minimal *In Vitro* Concentration = 1×10^6^ cells/ml
* In Vitro* Success (@ 24 h)>70%

*K = 100,000 x weight/(standard length)^3^

### Experiment 3 - In Vitro Fertilization Success Is Not Affected By Pooling Male Sperm

**Figure 3:**
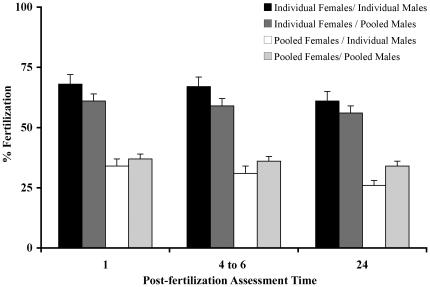
Pooling of gametes. The interactions of gametes during *in vitro* fertilization were assessed by examining combinations of individual and pooled gametes. Specifically, a clutch of eggs was divided into four parts for 4 treatments containing: 1) an individual female's oocytes and an individual male's sperm (N = 36 trials); 2) an individual female's oocytes and pooled sperm from at least 5 males (N = 58 trials); 3) pooled oocytes from 3 females and an individual male's sperm (N = 62 trials); and 4) pooled oocytes from 3 females and pooled sperm from at least 5 males (N = 49). Fertilization success was assessed at 1, 4 and 24 h. There was no affect on fertilization success using pooled male sperm (P>0.05) with individual female eggs, suggesting that there was no male/male interactions or effect that inhibited fertilization, but a 50% reduction in fertilization success for pooled female eggs was observed (P<0.05). The means were significantly different (P<0.05; ANOVA) between the groups that have different letters.

In order to establish high-throughput cryopreservation of sperm, pooling of male samples may be considered. In many resource centers and laboratories, pooling of female gametes is a relatively common process for *in vitro* fertilization. To assess whether these practices might affect fertilization success, combinations of individual and pooled gametes were used for *in vitro* fertilization (Fig. 3). There was no affect on fertilization success using pooled male sperm (P>0.05) with individual female eggs, suggesting that there was no male/male interactions or effect that inhibited fertilization, but a 50% reduction in fertilization success for pooled female eggs was observed (P<0.05). It was not clear what aspect of this process caused this reduction, such as female-female interactions during pooling, the time being held or dehydration prior to fertilization (eggs are held dry in covered Petri dishes). In the next experiment, the later two factors were analyzed.

### Experiment 4 - In Vitro Fertilization Success Is Not Affected By Pooling Female Oocytes

**Figure 4:**
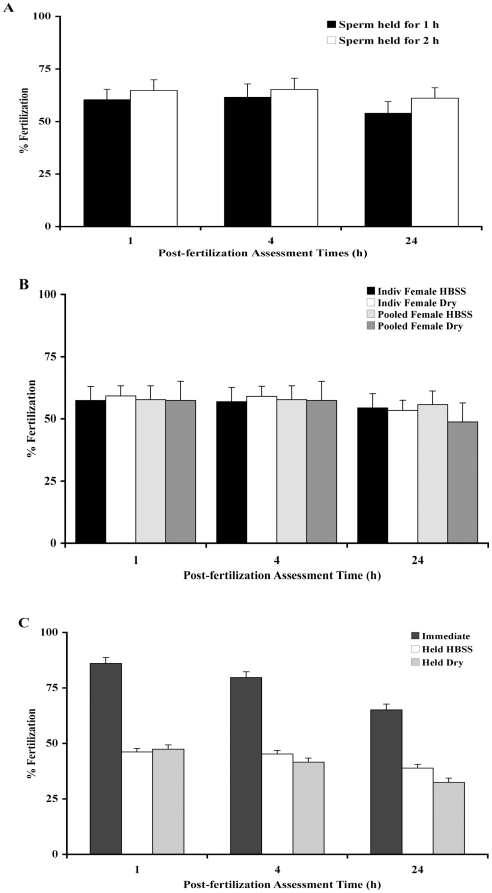
Factors reducing in vitro fertilization success. **A)** When pooled sperm samples from 5 males were held for 1 or 2 h on ice and used to fertilize eggs from freshly squeezed individual females (N = 10 *in vitro* trials/ time point), there was no difference in their fertilization success at 1, 4 and 24 h (P>0.05; ANOVA), suggesting that there was no male/male interactions or effect on holding the sperm that inhibited fertilization. The 1 h condition was considered the control condition, because it usually takes a while to collect the sperm and eggs prior to in vitro fertilization. **B)** To determine if dehydration was a factor affecting the eggs during fertilization, individual (N = 19) or pooled (N = 4) female eggs were held for 2 min in 40 µl of HBSS or kept dry, and then fertilized with pooled sperm (N = 10). There was no difference amongst the groups (P>0.05), suggesting that dehydration was not a factor in reducing fertilization success of individual or pooled eggs. **C)** To determine if holding time was a factor affecting fertilization success, a clutch of eggs from a single female was divided and either fertilized immediately or 5 min after being held dry or in HBSS. Fertilization success was affected by a holding time of 5 min, and (as shown in 4B) not by whether it was held dry or wet (P<0.05; ANOVA). The means were significantly different (P<0.05) between the groups that had different letters.

When pooled male samples were held for 1 or 2 h on ice and used to fertilize eggs from a freshly squeezed individual female, their fertilization success at 1, 4 and 24 h was not different (P>0.05; ANOVA), suggesting that the sperm holding process did not inhibit fertilization (Fig. 4A). To determine if dehydration is a factor affecting the eggs during fertilization, pooled male sperm was used to fertilize pooled or individual females eggs that were either put immediately into HBSS or kept dry for 2 min then fertilized (Fig. 4B). There was no difference between any of the groups (P>0.05), suggesting that how the eggs were held prior to fertilization was not a factor in reducing fertilization success. However, the fertilization success (∼55%) was slightly less than the 70 to 80 % mean fertilization success we often observed. So, to determine if holding time was a factor affecting fertilization success, a clutch of eggs from a single female was divided into three parts and one part was fertilized immediately, while the remaining two parts were fertilized 5 min after being held either dry or in HBSS (Fig. 4C). Again, holding the eggs dry or in HBSS did not matter (P>0.05), but the fertilization success was reduced 48% by a holding time of 5 min (P<0.05; ANOVA).

### Experiment 5 -In Vitro Fertilization Success Is Affected By Egg Handling

**Figure 5:**
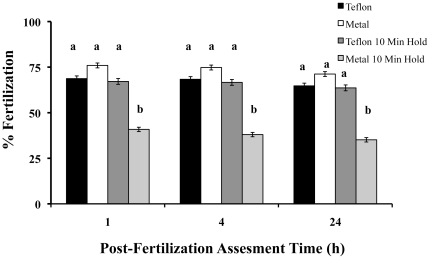
Factors increasing egg-holding time. To determine whether egg handling might have decreased the fertilization success observed in Fig. 4, eggs were handled with either the standard metal spatula or a Teflon-coated spatula. Eggs from individual females (N = 10) were initially divided in half with the Teflon-coated spatula, and then each half was divided again with either the Teflon-coated spatula or the metal spatula. Each quarter was moved into separate dish with the assigned spatula and covered in 40 µl of HBSS. One group of Teflon-moved and one group of metal-moved eggs were allowed to sit for 10 minutes before fertilization, while the remaining two groups were immediately fertilized by removing the HBSS and then fertilizing with pooled sperm (N = 5 males). Fertilization success was assessed at 1, 4 and 24 h. If the eggs were fertilized immediately, it did not matter what kind of tool was used to move the eggs, the fertilization success was unaffected (P>0.05). However, after moving the eggs with a metal spatula and waiting 10 min, there was a 50% loss in fertilization success. In contrast, there was no loss of fertilization success after moving the eggs with a Teflon-coated spatula and waiting 10 min (P<0.05; ANOVA). This suggested that a holding time of 10 min does not impact the fertilization success, and the reduction of fertilization success observed after a 5 min holding time in Experiment 4 was attributed to the egg handling.

Such a severe reduction (∼50%) in fertilization success after a 5 min holding time was contrary to what had been observed in many other fish species (Tiersch, pers. comm), therefore the egg-handling aspect of the *in vitro* process was examined to determine whether it was contributory to this loss. The use of metal spatulas to move and separate clutches of zebrafish eggs is a common practice in many laboratories and resource centers. If the eggs were fertilized immediately, it did not matter what kind of tool was used to move the eggs, the fertilization success was unaffected (P>0.05). After moving the eggs with a metal spatula and waiting 10 min, however, there was a 50% loss in fertilization success (Fig. 5). In contrast, there was no loss of fertilization success after moving the eggs with a Teflon-coated spatula and waiting 10 min (P<0.05). This suggested that a holding time of 10 min does not impact the fertilization success, and the reduction of fertilization success observed after a 5 min holding time in Experiment 4 was attributed to the egg handling (since metal spatulas were used to handle all eggs until experiment 5).

## Discussion

The results of this paper have generated recommendations to enhance the *in vitro* process throughout the zebrafish community and include: 1) using males of a body condition closer to 1.5 for maximal sperm concentration; 2) minimizing sperm wastage by using a working sperm concentration of 10^6^ motile cells/ml for fresh *in vitro* fertilization; and 3) never using metal or sharp-edged tools to handle the eggs prior to fertilization. All of these factors become important when considering high-throughput cryopreservation scenarios for zebrafish. These recommendations should be adhered to regardless of whether fresh or cryopreserved sperm is used. However, potentially a larger concentration of sperm must be cryopreserved to produce 10^6^ motile cells/ml in the dish when using thawed sperm.

Routine procedures have been developed in Dr. Tiersch's laboratory for sperm cryopreservation of oysters, and marine and freshwater fishes (e.g., [13]–[19]). Tiersch et al. have successfully cryopreserved the important, small-sized biomedical model, *Xiphophorus* (17–19), and their studies addressed the constraints for sperm cryopreservation of aquarium fish with small testes and sperm volumes [3], [20]. Huang et al. [17] obtained an average post-thaw sperm motility of 78±3% for *Xiphophorus*, and this targeted post-thaw incidence of fertilization success is likely achievable for zebrafish, as well. Some differences between the sperm of internally fertilizing fish and oviparous fish, like zebrafish, include: i) *X. helleri* sperm are transferred into the female in packets, instead of broadcast into the environment; ii) *X. helleri* sperm have a well-developed mitochondrial sheath in the midpiece, while external fertilizing sperm do not; iii) *X. helleri* sperm have thin, conical heads, while external fertilizing sperm have broad, spade-shaped heads; and most importantly, iv) *X. helleri* sperm maintain their motility for hours, while, once released into the environment, external fertilizing sperm are motile for only seconds.

Within the strict body conditions we set for the fish in these experiments, the males produced a consistent concentration of 10^10^ cells/ml, and the ideal concentration for fertilization success (using the methods described here) was a working concentration of 10^6^ cells/ml in the dish. This now becomes the unit that is needed for fresh fertilizations, and these data can be used to extrapolate for what is needed for cryopreserved sperm, as well. Cryopreservation often damages sperm, and more than likely a much higher concentration of cryopreserved sperm will be needed frozen in a straw or cryovial to achieve the final 10^6^ motile cells/ml needed for optimal fertilization for post-thaw sperm in the dish. Using the results from our work, ZIRC has preliminary results demonstrating a 3-fold increase in post-thaw fertilization success (Z. Varga, pers. comm.). This is a significant improvement in their process, just by understanding and then adjusting the reproductive parameters.

The greatest amount of variability observed in these experiments had to do with the holding time of the eggs prior to fertilization. The loss of oocyte viability after ovulation is relatively common in neotropical fish [21], and it may form one of the most challenging aspects for stock centers to control and standardize for *in vitro* fertilization. However, one of the major sources of this loss of *in vitro* fertilization success (50% after 10 min), stemmed from a common and seemingly innocuous practice used at ZIRC and other locations, whereby clutches of eggs were moved with metal spatulas. The sharp edges of the metal spatula may have nicked the surface of the chorion, causing damage, and thereby reducing fertilization success. The use of a Teflon-coated spatula maintained the same fertilization success throughout the 10 min test period. This is often the time needed for pooling clutches of eggs prior to *in vitro* fertilization.

However, to make the *in vitro* process even more efficient, maintaining eggs in an inactive state for at least 60 min would be desirable for high-throughput process and might be accomplished by additives to the buffers [22]. In fact, some trout ovulatory proteins act as protease inhibitors and are responsible for maintaining oocytes in an inactivated state [23]. These types of ovarian fluid extenders have been tried in zebrafish. Sakai et al. [24] reported that bovine serum albumin maintained inactive zebrafish oocytes for 1 h. Corley-Smith et al. [25] extended this timeframe to 6 h in zebrafish using Coho salmon (*Oncorhynchus kisutch*) ovarian fluid. Seki et al [26] reported that zebrafish oocytes matured in 90% Leibovitz L-15 medium at pH 9.0 with bovine serum albumin increased the duration of fertilization ability of oocytes. It may be that the bovine serum albumin provides suitable substrate for proteolysis and holding the eggs at pH 9.0 may further inactivate the enzymes, thereby extending the holding time. The problem is trying to standardize some of these procedures for stock centers so that the variability in the fertilization success can be reduced and that the additives are standardized and easily purchased. However, these processes may be worthwhile incorporating into the *in vitro* fertilization process and are currently under study.

This paper is part of an ongoing collaboration funded by the National Institutes of Health, National Center for Research Resources to help improve high-throughput resource preservation for aquarium fish. Findings from our studies have already provided the scholarly information necessary to significantly improve the ability to preserve and safeguard the diversity of zebrafish strains used in biomedical research. Without this approach, the zebrafish community will continue to struggle with low and variable capacities to protect these thousands of valuable genotypes. With appropriate fundamental and applied data it will be possible to begin systematic germplasm cryopreservation to significantly improve management efficiency (including reducing cost) to maintain the NIH resource.

In addition, ZIRC has entered an agreement for a zebrafish back-up repository with USDA National Animal Germplasm Program in Fort Collins, Colorado (www.ars-grin.gov/animal/). The USDA repository was built to withstand extraordinary weather conditions, and has ample storage capacity. The National Animal Germplasm Program is responsible for maintaining (through cryopreservation) all agriculturally important germplasm, and as with zebrafish, cryopreservation of cultured fish species is not standardized and yields inconsistent results. The high-throughput platform for zebrafish cryopreservation that we are developing will be a robust model that will be applicable to other aquatic species. This type of federal partnering will become more important as stock centers struggle to manage the rapid increase in genetic resources and may benefit many aspects of national genetic repositories.
